# *GhVIM28*, a negative regulator identified from VIM family genes, positively responds to salt stress in cotton

**DOI:** 10.1186/s12870-024-05156-8

**Published:** 2024-05-21

**Authors:** Zhining Yang, Xuke Lu, Ning Wang, Zhengding Mei, Yapeng Fan, Menghao Zhang, Lidong Wang, Yuping Sun, Xiao Chen, Hui Huang, Yuan Meng, Mengyue Liu, Mingge Han, Wenhua Chen, Xinrui Zhang, Xin Yu, Xiugui Chen, Shuai Wang, Junjuan Wang, Lanjie Zhao, Lixue Guo, Fanjia Peng, Keyun Feng, Wenwei Gao, Wuwei Ye

**Affiliations:** 1 Institute of Cotton Research of Chinese Academy of Agricultural Sciences / Zhengzhou Research Base, State Key Laboratory of Cotton Bio-Breeding and Integrated Utilization, School of Agricultural Sciences, Zhengzhou University / National Center of Technology Innovation for Comprehensive Utilization of Saline-Alkali Land, Anyang, Henan 455000 China; 2 Engineering Research Centre of Cotton, Ministry of Education / College of Agriculture, Xinjiang Agricultural University, 311 Nongda East Road, Urumqi, 830052 China; 3 Institute of Crop Sciences, Gansu Academy of Agricultural Sciences, Lanzhou, Gansu 730070 China; 4 Hunan Institute of Cotton Science, Changde, Hunan 415101 China

**Keywords:** E3 ubiquitin ligase, *GhVIM28*, Salinity stress, Antioxidant

## Abstract

The VIM (belonged to E3 ubiquitin ligase) gene family is crucial for plant growth, development, and stress responses, yet their role in salt stress remains unclear. We analyzed phylogenetic relationships, chromosomal localization, conserved motifs, gene structure, *cis*-acting elements, and gene expression patterns of the VIM gene family in four cotton varieties. Our findings reveal 29, 29, 17, and 14 members in *Gossypium hirsutum (G.hirsutum), Gossypium barbadense (G.barbadense), Gossypium arboreum (G.arboreum), and Gossypium raimondii (G. raimondii)*, respectively, indicating the maturity and evolution of this gene family. motifs among *GhVIMs* genes were observed, along with the presence of stress-responsive, hormone-responsive, and growth-related elements in their promoter regions. Gene expression analysis showed varying patterns and tissue specificity of *GhVIMs* genes under abiotic stress. Silencing *GhVIM28* via virus-induced gene silencing revealed its role as a salt-tolerant negative regulator. This work reveals a mechanism by which the VIM gene family in response to salt stress in cotton, identifying a potential negative regulator, *GhVIM28*, which could be targeted for enhancing salt tolerance in cotton. The objective of this study was to explore the evolutionary relationship of the VIM gene family and its potential function in salt stress tolerance, and provide important genetic resources for salt tolerance breeding of cotton.

**Supplementary Information:**

The online version contains supplementary material available at 10.1186/s12870-024-05156-8.

## Introduction

The post-translational modification of proteins by ubiquitination plays a critical role not only in human health but also in the regulation of plant growth, development, and responses to various environmental stress [1–4]. Plants are continuously exposed to adverse growth conditions throughout their lifecycle. Ubiquitination, by modulating protein abundance and activity, plays a pivotal role in orchestrating the transcriptional changes necessary for adapting to non-biological stressors. Plant growth and development are profoundly impacted by the regulatory control exerted by ubiquitin-mediated protein stability. The ubiquitin–proteasome system (UPS) operates within the cytoplasm and nucleus, regulating protein levels and eliminating potentially accumulated misfolded or impaired proteins caused by exposure to abiotic stress [5]. The attachment of ubiquitin to its substrates requires the sequential involvement of three enzymes, which are crucial constituents of the ubiquitin conjugation system [6]. The initial stage of this process involves ubiquitin activation, which occurs through a two-step reaction catalyzed by the ubiquitin-activating enzyme (E1) and necessitates ATP. The catalytic activity of E1 results in the formation of a thioester bond between the glycine 76 residue of ubiquitin and the active cysteine site of E1. Subsequently, the E1-ubiquitin complex interacts with the ubiquitin-conjugating enzyme (E2), facilitated by conformational alterations that promote the proximity of their respective active sites, thereby facilitating the transfer of ubiquitin from one enzyme to another [7]. The ultimate step of this process entails the reciprocal interaction between the E2-ubiquitin complex and the E3 ubiquitin ligase, resulting in the transfer of ubiquitin onto the substrate. E3 assumes the responsibility of discerning the definitive substrate for ubiquitination and facilitates the formation of a peptide bond between the C-terminal glycine 76 of ubiquitin and the amino group of the substrate’s lysine residue [7].

The UPS comprising ubiquitin (Ub), E1, E2s, E3s, 26S proteasome, and target proteins, represents a rapid regulatory mechanism for the selective degradation of proteins in plants, playing a pivotal role in growth and development. A mounting body of evidence supports the significance of UPS as an essential component of plant responses to environmental stresses. These stresses include, but are not limited to, drought, salinity, cold, nutrient deprivation, and pathogen attack. The UPS functions as the principal pathway for protein degradation, accounting for the degradation of a substantial proportion of cellular proteins (80%-90%) [8]. The adaptability of a plant’s proteome plays a pivotal role in its ability to withstand non-biological stresses, including to salinity, radiation, heavy metal toxicity, nutrient deficiency, cold, and drought. The UPS confers upon plants the capability to modulate their proteomes, thereby allowing them to sense and respond to environmental pressure with enhanced efficacy and efficiency. The specificity within the UPS is primarily mediated by E3 ubiquitin ligases, which are instrumental in substrate recognition and subsequent ubiquitination. Nonetheless, the substrate repertoire of numerous E3 ubiquitin ligases, especially the precise molecular characteristics they recognize, remains largely uncharted. E3 ubiquitin ligases, functioning as the ultimate enzymatic entity within the UPS, display multifarious functionalities. Their indispensable role in governing the specific degradation of proteins highlights their pivotal importance in cellular processes [9].

Research has demonstrated the critical role of E3 ubiquitin ligases in orchestrating plant responses to stress conditions [10]. Ubiquitin, a highly conserved protein involved in various cellular processes, contains seven conserved lysine residues (Lys6, Lys11, Lys27, Lys29, Lys33, Lys48, and Lys63). These lysine residues play pivotal roles in generating structurally diverse polyubiquitin chains, thereby regulating distinct functions within cells [11]. High temperatures induce tobacco [12], Potato [13] and Corn [14] in the expression of multiple polyubiquitin genes. Indeed, overexpression of a single monoubiquitin gene enhances tolerance to multiple stresses without adversely affecting growth and development under favorable conditions [12]. Little is known about the mechanism of E3 ubiquitin ligase in cotton in response to salt stress, which limits our ability to use this mechanism to explore plant resilience. Due to the abundance of RING E3 ligases and their unique functions in plant developmental processes and stress response, RING E3 ligases have been increasingly studied in recent years [15].

The VIM protein is an E3 ubiquitin ligase [16]. Camille et al. elucidated that plant long non-coding RNAs (lncRNAs) coordinate Polycomb-mediated gene silencing and DNA methylation by interacting with *VIM1* [17]. Additionally, plant E3 ligases play a major role in the response to abiotic stresses [18]. For instance, studies have shown that *PUB* genes are crucial in regulating salt stress, potentially serving as targets for breeding salt-tolerant sorghum (*Sorghum bicolor* L.) in the future [19]. Furthermore, research has demonstrated that the C3HC4-type E3 ligase (OsRFPHC-4) contributes to improving salt tolerance and maintaining Na^+^ / K^+^ homeostasis by regulating changes in Na^+^ / K^+^ transporters [20]. On the contrary, *GmPUB21* negatively regulates drought and salinity tolerance by increasing stomatal density and aperture through the ABA signaling pathway [21]. In contrast, the OsSIRH2-14 RING E3 ligase positively regulates the salinity stress response by modulating the stability of salt-related proteins [22]. Studies show that *TaPUB1* plays an essential role in salt tolerance in wheat (*Triticum aestivum* L.) [23]. VARIANT IN METHYLATION 1 (*VIM1*), *VIM2*, and *VIM3* are orthologous to mammalian UBIQUITIN-LIKE, CONTAINING PHD AND RING FINGER DOMAINS 1 (UHRF1) and have been shown to regulate CG methylation [24]. *VIM1*, *VIM2*, and *VIM3* have overlapping functions in maintenance of global CpG methylation and epigenetic transcriptional silencing [25]. The study found that CG methylation was significantly reduced in vim1, vim2, and vim3 (vim1 / 2 / 3), resembling *met1*. Importantly, individual vim1, vim2, and vim3 did not affect CG methylation, indicating complete functional redundancy in regulating CG methylation [26].

In this study, the evolutionary characteristics and potential function of VIM gene family were identified. In addition, conserved motifs, chromosomal distribution, *cis*-acting elements of promoters and expression patterns under different stresses in *G. hirsutum* were also analyzed. Then, a highly expressed gene *GhVIM28* was selected to study its role in salt stress. Our results provide an identifiable basis for further study of the mechanism of VIM gene family genes, and the biological function of *GhVIM28* in response to salt stress.

## Material and methods

### Identification and evolutionary analysis of VIM protein family members

Database Source and Family Member Identification.

The genome annotation files and protein files of *G. hirsutum*, *G. barbadense*, *G. arboreum*, and *G. raimondii* were obtained from the Cotton Functional Genomics Database (CottonFGD) website (https://cottonfgd.net) [27], Database Source and Family Member Identification.

The genome and protein files of *Arabidopsis thaliana* were obtained from the TAIR10 database (http://www.arabidopsis.org/). The protein sequences of five VIM family members, as reported by *Arabidopsis* VIM1 (At1G57820), VIM2 (At1G66050), VIM3 (At5G39550), VIM4 (At1G66040), VIM5 (At1G57800), were employed to identify relevant family members through the utilization of local BLAST software and commands. Subsequently, the identified genes underwent structural domain identification using Pfam and SMART online databases. Moreover, the identified genes were further assessed using Batch CD-Search accessible on the NCBI website [28], and genes with incomplete C and N termini were manually deleted. In this study, the *VIMs* genes of *G. hirsutum*, *G. barbadense*, *G. arboreum*, and *G. raimondii*, and *A. thaliana* (At) were renamed according to the chromosomal location where they are located (Table 1). In this study, various biophysical and biochemical characteristics of *VIM* genes from four cotton species were further retrieved using CottonFGD (https://cottonfgd.org/), including transcript length, exon / intron length, protein length, molecular weight, isoelectric point, total average of hydrophilicity and charge (Table 1) [27].

**Table 1:** Physicochemical properties of upland cotton gene family members

New ID	Gene ID	Protein Length (aa)	Molecular Weight (KDa)	Isoelectric Point	Subcellular localization prediction
*GhVIM1*	GH_A03G1752	990	111.950	9.832	nucleus
*GhVIM2*	GH_A05G2877	657	72.892	8.371	nucleus
*GhVIM3*	GH_A05G3503	701	78.068	8.986	chloroplast
*GhVIM4*	GH_A05G4179	664	73.334	7.311	nucleus
*GhVIM5*	GH_A08G1897	1038	115.708	5.309	nucleus
*GhVIM6*	GH_A09G0416	366	41.300	9.179	nucleus
*GhVIM7*	GH_A09G1909	659	73.411	7.322	nucleus
*GhVIM8*	GH_A09G2516	693	77.642	5.268	nucleus
*GhVIM9*	GH_A11G0036	685	76.314	6.895	nucleus
*GhVIM10*	GH_A11G3484	697	76.964	7.219	nucleus
*GhVIM11*	GH_A12G2136	683	76.266	8.036	nucleus
*GhVIM12*	GH_A12G2339	919	103.087	8.096	nucleus
*GhVIM13*	GH_A12G2885	656	73.189	7.553	nucleus
*GhVIM14*	GH_A13G0038	667	74.096	8.191	nucleus
*GhVIM15*	GH_D02G1908	996	112.611	9.893	nucleus
*GhVIM16*	GH_D03G1208	879	98.942	5.036	nucleus
*GhVIM17*	GH_D04G0198	664	73.042	7.311	nucleus
*GhVIM18*	GH_D04G0755	701	77.965	8.925	nucleus
*GhVIM19*	GH_D05G2897	657	72.759	8.454	nucleus
*GhVIM20*	GH_D08G1911	1022	113.689	5.105	nucleus
*GhVIM21*	GH_D09G0383	366	41.305	9.175	nucleus
*GhVIM22*	GH_D09G1862	658	73.103	7.193	nucleus
*GhVIM23*	GH_D09G2452	693	77.748	5.472	nucleus
*GhVIM24*	GH_D11G0041	683	76.074	6.730	nucleus
*GhVIM25*	GH_D11G3490	697	76.636	6.803	nucleus
*GhVIM26*	GH_D12G2138	668	74.576	8.134	nucleus
*GhVIM27*	GH_D12G2354	919	103.146	7.919	nucleus
*GhVIM28*	GH_D12G2908	665	74.179	7.304	nucleus
*GhVIM29*	GH_D13G0035	667	74.160	8.282	nucleus

### Sequences alignments and phylogenetic analysis

The alignment of protein sequences and the subsequent construction of the phylogenetic tree were carried out utilizing the MEGA7.0 software. To construct the phylogenetic tree, the TBtools software (Version 1.098693) was utilized, employing the maximum likelihood (ML) method, with bootstrap values estimated from 5000 replicates [29].

### Chromosomal localization of *GhVIMs*

The gene annotation file (in GFF3 format) for upland cotton was obtained from CottonFGD (https://cottonfgd.org/). The visualization of the physical position of the *VIM* gene on the chromosomes of the upland cotton genome was achieved using the Toolbox for Biologists software (TBtools, version 1.098693) [29].

### Prediction of *GhVIMs* conserved protein motifs and gene structure

In this study, the online web tool Multiple Em (https://meme-suite.org/meme/tools/meme) was employed for Motif Elimination (Ver. 5.4.1) to predict the conserved protein motifs of VIM proteins [30]. The phylogenetic tree, conserved motifs, and gene structure diagrams of *GhVIMs* were constructed using TBTools (version 1.098693) based on the NWK file of the phylogenetic tree, the gff3 file of upland cotton, and the MAST file obtained from the MEME website (version 5.4.1) [29].

### Promoter region analysis of *GhVIMs*

In this study, the DNA sequence of the upstream 2000 bp region of *GhVIM* was downloaded from CottonFGD (https://cottonfgd.org) as the promoter region. The online website PlantCARE (http://bioinformatics.psb.ugent.be/webtools/plantcare/html/) was utilized to predict *cis*-acting elements in the *GhVIMs* gene promoter region. Specifically, *cis*-acting elements associated with light response, plant growth and development, plant hormones, and abiotic stress were selected for further analysis.

### Analysis of gene expression patterns of *GhVIMs*

The public RNA-seq data from previous research (PRJNA490626) [31] were used to analyze the expression profiles of *GhVIMs* in cotton under abiotic stresses, and in different tissues (http://cotton.zju.edu.cn/index.htm).

### qRT-PCR of *GhVIMs*

The response of cotton leaf tissues to salt (200 mM NaCl), abiotic stresses at different time intervals (0 h, 6 h, 12 h and 24 h) was analyzed by qRT-PCR. Seeds of Zhong 9807 were sown in medium and incubated in a light incubator at 25 °C (16 h / 8 h day / night) until the three-leaf stage. Salt stress treatments were carried out and sampled at the three-leaf stage of cotton, and three independent biological replicates were used for the experiment. In this study, total RNA was extracted from cotton samples using FastPure Universal Plant Total RNA Isolation Kit (RC411-01) (Vazyme Biotech Co., Ltd), and then analyzed according to the Trans Start Top Green qPCR Super Mix (Trans Gene Biotech Co. Beijing, China) instruction manual. 29 primers for *GhVIMs* were designed on the online website The Gen Script online tool (https://www.genscript.com), and all primer sequences are shown Supplementary Table S1 RT-qPCR experiments were performed in Bio-Rad 7500 rapid fluorescence quantitative PCR platform. Finally, the relative expression of *GhVIMs* genes was calculated using the 2^−ΔΔCt^ method [32]. Primer sequences are listed in Supplementary Table S1.

### Virus induced gene silencing (VIGS) experiment

A 300 bp silencing fragment was designed using SGN-VIGS (https://vigs.solgenomics) for *GhVIM28* gene. Primers for amplification of the 300 bp silencing fragment of *GhVIM28* gene were designed using SnapGene software (GhVIM28-VIGS-F: GCCTCCATGGGGATCCAAATGTGCTCCATTGTCCTT, GhVIM28-VIGS-R: CGAGACGCGTGAGCTCGTATCCACTTTCATCCTCTT) and their specificity was validated using NCBI website.

The upland cotton raw material used in the experiment was Zhong 9807, which was from the Cotton Research Institute of the Chinese Academy of Agricultural Sciences. The land cotton variety Zhong 9807 was cultivated in nutrient soil and then placed in an incubator with a light / dark cycle of 16 h / 8 h, 50% ambient humidity, and a temperature of 25 °C / 20 °C (light / dark). Agrobacterium was injected into cotton plants at the time of unfolding of both cotyledons, and the treatment occurred at the two-leaf, one-heart stage of cotton. After ligating the fragments into the pYL156 vector and transforming into Agrobacterium, pYL156:*GhVIM28*, pYL156:PDS, and pYL192 were cultured to OD600 = 1.2–1.5 [33] (pYL156 carrier information is available in Supplementary Figure S2). Each mixture was injected into the lower side of the cotyledons of *G. hirsutum* material Zhong 9807. After injection, seedlings were placed in the dark for 24 h, followed by a 16 h light / 8 h dark cycle at 25 °C / 20 °C (light / dark). Cotton seedlings were treated with 200 mmol/L NaCl solution for 24 h, which occurred at the two-leaf, one-heart stage of cotton. Subsequently, the leaves were used as samples, quickly frozen with liquid nitrogen and stored in a -80 °C refrigerator for subsequent experiments.

### Determination of physiological and biochemical indexes

0.1 g of fresh leaves were taken to determine the content or enzyme activity of each substance in the plants using Proline (PRO) Content Assay Kit (Nanjing Jiancheng Institute of Biological Engineering, A107-1-1), Malondialdehyde (MDA) Assay Kit (Nanjing Jiancheng Institute of Biological Engineering, A003-3-1) and Superoxide Dismutase (SOD) Activity Assay Kit (Beijing Solarbio Science & Technology Co., Ltd., BC0170), respectively. Three biological replicates were available for each sample. The DAB staining method called diaminobenzidine method was used to detect the active site of peroxidase in cells. Three leaves each of pYL156 and pYL156: *GhVIM28* were taken after NaCl stress and placed in DAB solution, darkened for 12 h, and observed after decolorization with 95% ethanol. The dark brown polymerization products represent the reaction of DAB with hydrogen peroxide. For trypan blue staining, the staining solution was first prepared according to the propor-tions of 10 mL lactic acid, 10 mL glycerol, 10 g phenol, 10 mg trypan blue (Solarbio, Beijing, T8070) and 10 mL distilled water, then soaked plant leaves in this staining solution, bath in a boiling water for 2 min, cooled at room temperature, and decolorized in chloral hydrate (1.25 g / mL). Daily replacement of decolorization solution was performed until the background color is eliminated.

## Results

### Identification of VIM proteins

In order to identify members of the VIM gene family in cotton, we used five VIM protein sequences from *A. thaliana* as references. We performed a local BLAST search in the databases of four cotton species, including *G. hirsutum*, *G. barbadense*, *G. raimondii*, and *G. arboreum*, with an E-value threshold set at E > 10^–5^. The identified members of the VIM gene family were then validated using the NCBI-CDD database to confirm the presence of complete domains, and incomplete members lacking N-terminal or C-terminal domains were manually removed. The resulting family members were named based on their chromosomal locations. A total of 29, 29, 17, and 14 members were identified in *G.hirsutum, G.barbadense, G.arboreum, and G.raimondii*. From the final identification results, the ratio of *VIM* genes in allopolyploid *G.hirsutum*, *G.barbadense* was close to 1:1, while the ratio in diploid *G.arboreum* and *G.raimondii* was also close to 1:1. Additionally, the ratio of *VIM* genes in allopolyploid and diploid cotton was close to 2:1, which is consistent with the evolutionary selection of allopolyploid cotton through hybridization of two diploid cotton species. To further analyze the biophysical properties of the *VIM* genes in these four cotton species, including subcellular localization, protein length, molecular weights (MWs), and isoelectric points (pI), we focused on upland cotton, which is the most widely cultivated polyploid model plant. The biophysical properties of the 29 *VIM* genes in upland cotton were analyzed (Table 1). The protein lengths of these family members ranged from 366 (*GhVIM1*) to 1038 (*GhVIM29*) amino acids. The predicted pI ranged from 5.036 to 9.893, and MW ranged from 41.300 kDa to 115.708 kDa. Subcellular localization predictions showed that the protein encoded by *GhVIM3* was predicted to localize in the chloroplast, while the rest of the VIM family proteins were predicted to localize in the nucleus. This may be determined by specific domain characteristics of this family.

### Phylogenetic tree of *VIM* genes of four cotton species

**Fig. 1:**
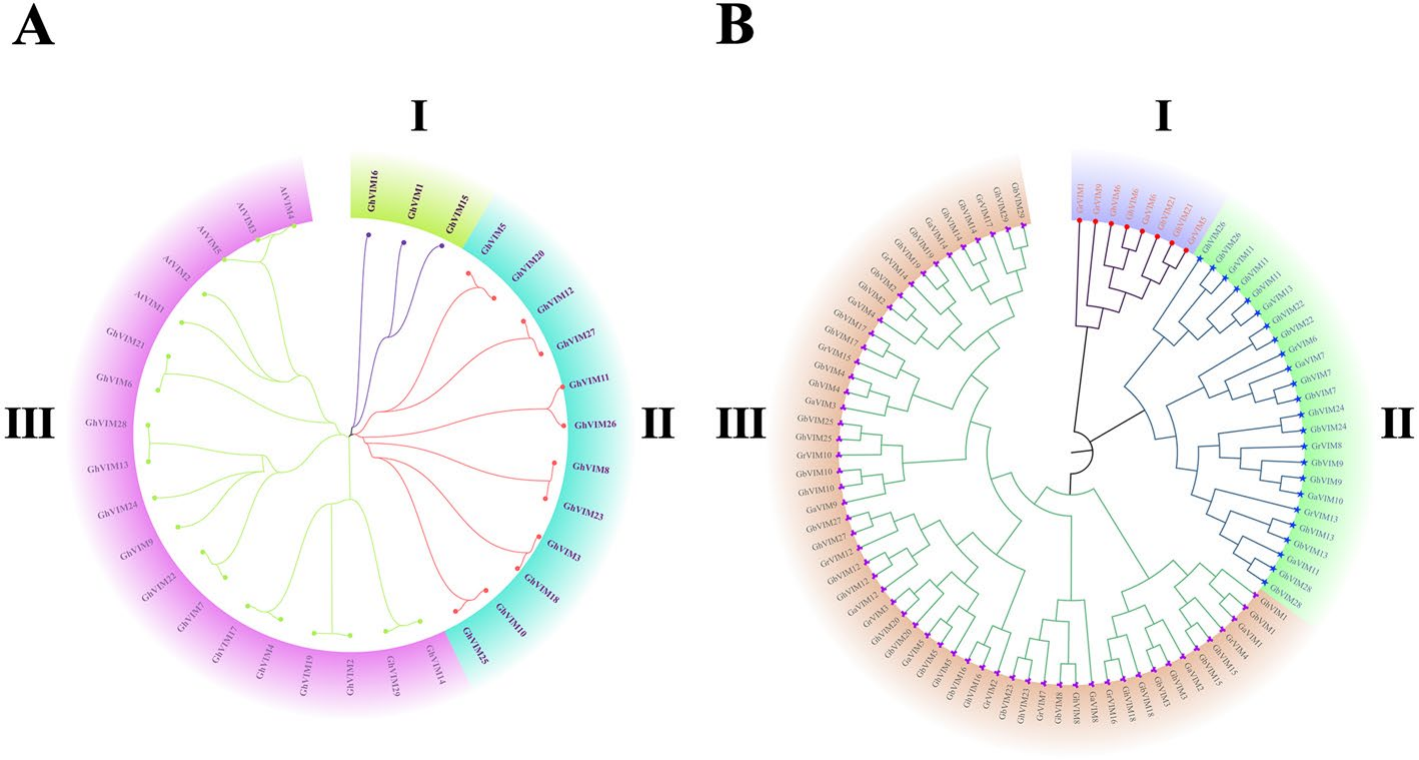
VIM gene family phylogenetic tree in five plant species. **A** Phylogenetic relationship of 34 identified *VIM* genes from *G.hirsutum* and *A.thaliana*. **B** Phylogenetic relationship of 89 identified *VIM* genes from four cotton species

The identified members of the VIM gene family were used to construct an evolutionary tree in MEGA software and saved as a Newick file format. Subsequently, the evolutionary tree was visually enhanced using the EvolView online tool (Fig. 1). To understand the evolutionary history of the *VIM* gene in the five plants, 34 VIM protein sequences (29 from *G.hirsutum* and 5 from *A.thaliana*) were compared using the CLUSTALX software, and a rootless phylogenetic tree was constructed using the MEGA7 Maximum Likelihood method (ML) of MEGA7 (Fig. 1A) [34].

The VIM gene family members in the four major cotton species were classified into three subgroups, namely Group I, Group II, and Group III. Through analysis of these three subgroups, it was observed that *VIM* genes in cotton are predominantly found in Group III, which represents the majority of *VIM* genes in upland cotton (Fig. 1B). The overall gene counts remain relatively stable among the different cotton species, regardless of whether they are from the tetraploid A and D sub genomes or the diploid genome.

### Chromosomal localization of the *G. hirsutum* VIM gene family

**Fig. 2:**
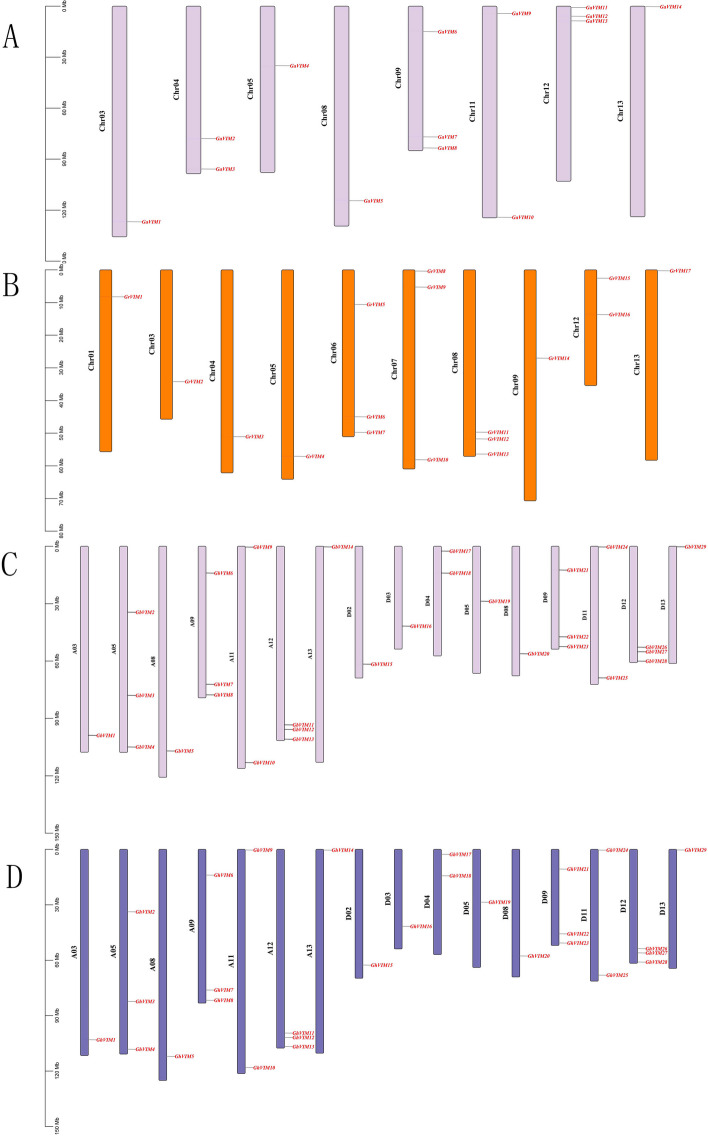
Chromosomal positions of *VIM* genes in *G. arboreum*, *G. raimondii*, *G. barbadense*, and *G. hirsutum*. The chromosomal positions of *VIM* genes in *G. arboreum* (**A**), *G. raimondii* (**B**), *G. barbadense* (**C**), and *G. hirsutum* (**D**)

In order to investigate the genetic variation and chromosomal association of the VIM gene family, this study conducted chromosomal mapping of all *VIM* genes. A total of 89 cotton *VIM* genes were found to be distributed on the chromosomes of four cotton species (Fig. 2). The results revealed that the second chromosome of *G. arboreum* harbored the highest number of *GaVIM* genes, while the sixth, seventh, and eighth chromosomes of *G. raimondii* contained the highest number of *GrVIM* genes. All 29 *VIM* genes in *G. hirsutum* and *G. barbadense* were assigned to their respective chromosomes. Among them, 14 *VIM* genes were located in At subgenome, and 15 *VIM* genes were located in Dt subgenome. The chromosomal distribution of *VIM* genes in *G. hirsutum* and *G. barbadense* exhibited similarity, indicating the evolutionary maturity of the VIM gene family. Additionally, tandem repeat events were observed, with two or three adjacent genes forming gene pairs during the evolution of the VIM gene family as shown in Fig. 2.

### Conserved motif and gene structure analysis of *G. hirsutum VIM* gene

**Fig. 3:**
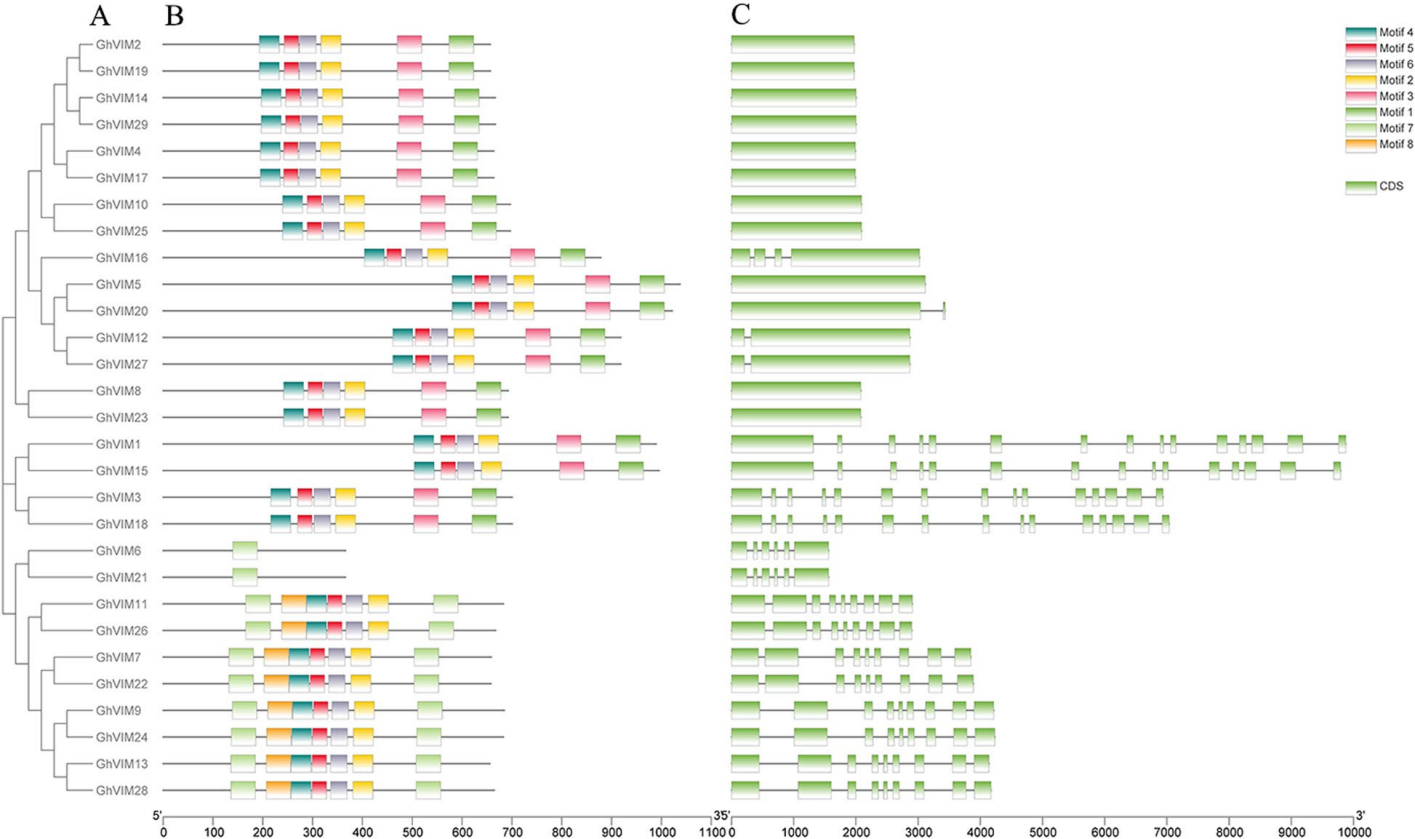
Phylogenetic tree, conserved motifs and exon–intron organization of *GhVIMs* genes from *G. hirsutum*. **A** Phylogenetic tree of *GhVIM* genes; **B** Conserved motifs of GhVIM proteins; **C** Exon–intron structures of *GhVIM* genes

In plants, genes consist of two parts, coding and non-coding regions, which include exons and introns. The structure and arrangement of introns and exons can be used to analyze the evolutionary relationships among different gene members. Previous studies have shown that the distribution pattern of exons and introns in genes is related to their biological function [35]. Usually, genes within the same subfamily exhibit similar intron–exon arrangements in terms of intron numbers and exon lengths. In this study, using upland cotton as an example, the relationship between conserved motifs and gene structures of the *VIM* gene in cotton was analyzed. A phylogenetic tree and gene structure diagram of upland cotton were constructed (Fig. 3). The results showed that the distribution of exon regions varied from 1 to 15 in *GhVIMs* genes, while *GhVIMs* genes within the same evolutionary branch exhibited similar intron–exon structures and arrangements in terms of intron numbers and exon lengths (Fig. 3A, C). Among the 29 *GhVIMs* genes, approximately 37.93% (11 genes) had no introns, about 10.34% (3 genes) had only one intron, and 51.72% (15 genes) had two or more introns (Fig. 3C). The presence of multiple introns in *GhVIMs* gene members may be attributed to gene duplication and evolution within the gene family, leading to intron duplication and diversity. Multiple introns provide opportunities for gene splicing, and the diversity of gene splicing can enhance gene functionality and regulatory complexity, thereby resulting in different gene characteristics in different tissues and developmental stages. In summary, this study revealed a strong correlation between the gene structure and phylogeny of *GhVIMs* genes, indicating a conserved pattern of gene structure.

To better understand the evolutionary relationship among *GhVIMs* gene members, a phylogenetic tree of *GhVIMs* genes (Fig. 3A) and the distribution of protein motifs (Fig. 3B) were generated. The results showed that each GhVIM protein possessed various conserved motifs ranging from 1 to 8. *GhVIMs* genes with the same distribution pattern of protein motifs were clustered together within the same evolutionary branch and adjacent to each other. Different evolutionary branches exhibited unique distribution patterns of conserved motifs. Motif was present in all branches of the evolutionary tree. The protein sequence at the C-terminus of *GhVIMs* was more conserved than the N-terminus sequence. Overall, this study found that *GhVIMs* genes also have a strong relationship between gene structure and protein motif distribution on an evolutionary basis and exhibit conserved patterns of gene structure and protein motif.

### Gene expression and *cis*-acting element analysis of *GhVIMs*

**Fig. 4:**
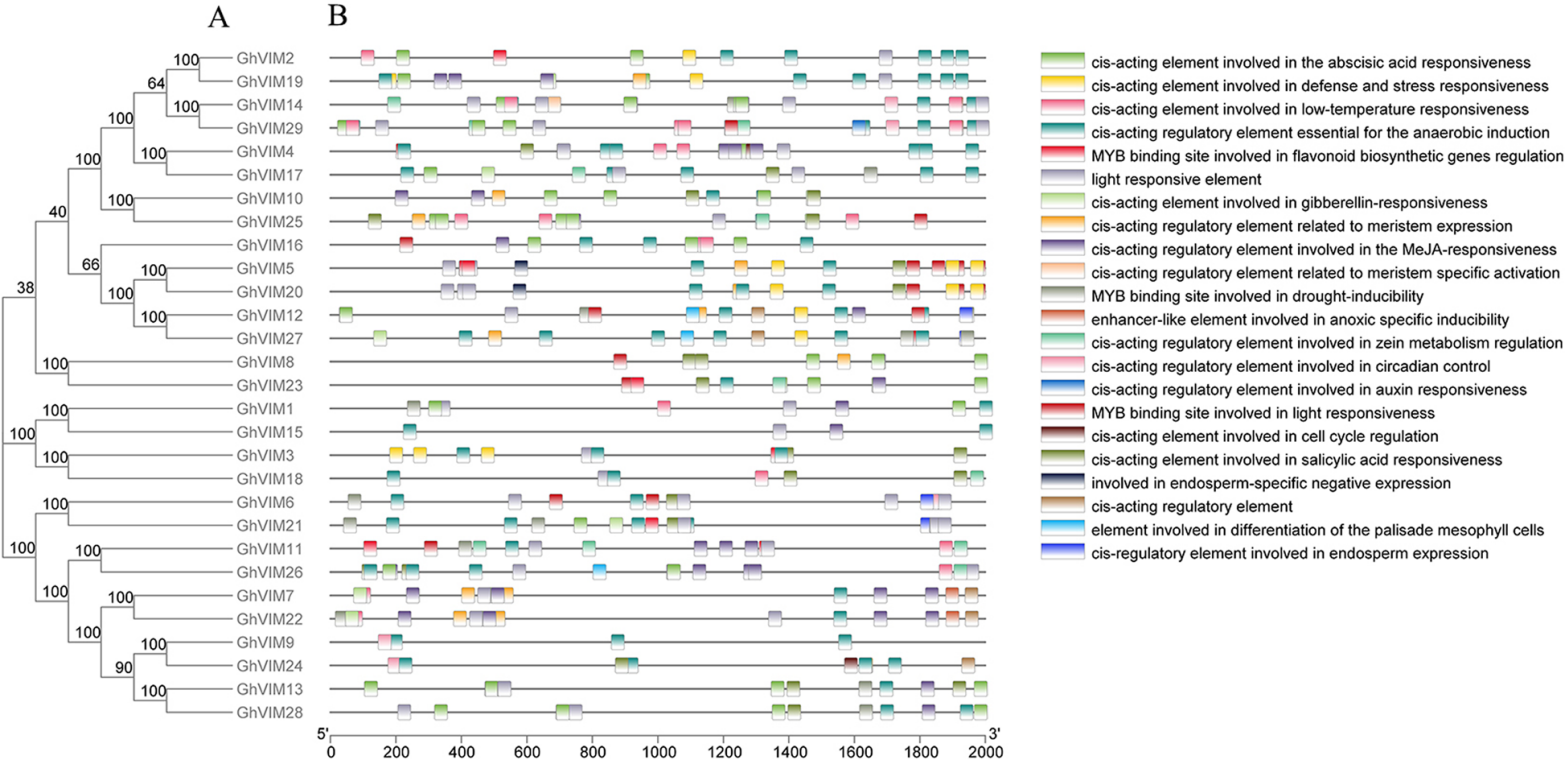
Analysis of *cis*-acting elements in the promoter region of *GhVIMs* genes family. **A** Evolutionary relationship of *GhVIMs* genes; **B** Prediction of *cis*-acting elements of *GhVIMs* genes promoter

*Cis*-acting elements are sequences located in the vicinity of a gene that can influence gene expression and are mainly involved in the regulation of gene expression. *Cis*-acting elements generally include enhancers, promoters, and inducible elements, among others. The different combinations and sequence characteristics of these elements determine the expression pattern and regulation mode of genes. We analyzed the promoter regions of the 29 *GhVIMs* in *G. hirsutum*, which mainly included the DNA sequences upstream of the transcription start site (TSS) by 2000 bp. In the promoter regions of the *GhVIM* genes, we identified numerous *cis*-acting elements involved in stress responses, such as *cis*-acting elements involved in low-temperature response, defense and stress response, and MYB *cis*-acting elements induced by drought (Fig. 4).

Furthermore, we found differences in the *cis*-acting elements of *GhVIMs* from different evolutionary branches. The MYB transcription factor also participates in and regulates plant responses to non-biological stresses such as drought, ultraviolet radiation, cold stress, high temperature stress, and salt stress. In transgenic plants, overexpression of MYB12 significantly increases flavonoid accumulation, enhancing plant tolerance to non-biological stresses such as drought and oxidative stress [36]. We identified numerous *cis*-acting elements of MYB transcription factors associated with stress response, which were widely distributed across all evolutionary branches.

The plant hormone response category contains three types of *cis*-acting elements, and similarly, *cis*-acting elements related to growth and development categories were also identified. It is evident that *cis*-acting elements associated with stress, hormone response, and growth and development are abundantly found in the *GhVIMs* genes of upland cotton, indicating their crucial roles in plant growth and development, plant hormone responses, and various stress conditions.

**Fig. 5:**
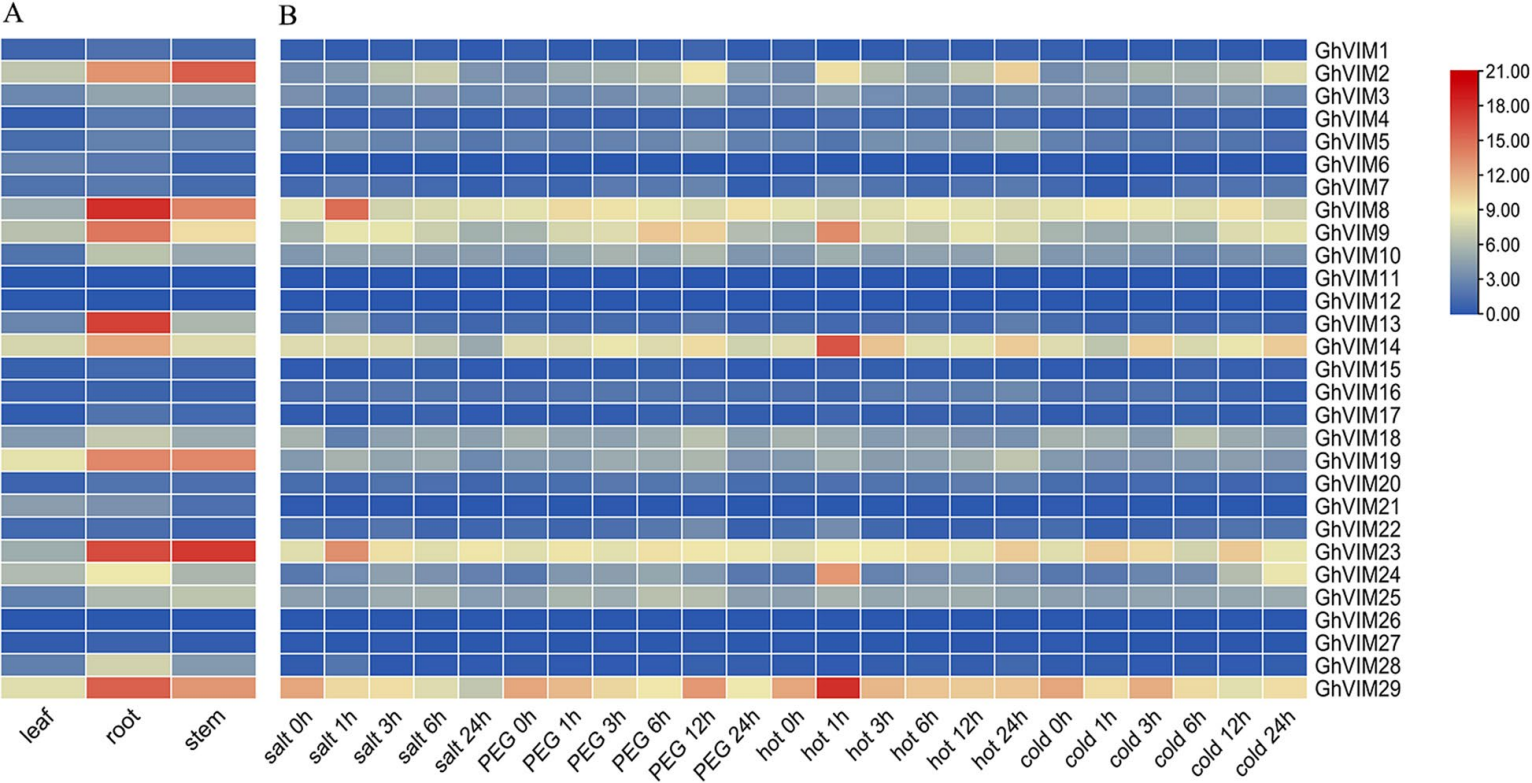
Analysis of the expression patterns of 29 genes in the GhVIMs genes family. **A** The *GhVIM* genes exhibit distinct expression patterns in response to cold, heat, salt, and PEG stress. **B** *GhVIM* genes tissues specific expression patterns. The change from blue to red represents the change in gene expression level from low to high

The expression profiles of *GhVIMs* in cotton and different tissues under abiotic stress were analyzed applying the differential expressed genes (DEGs) from the transcriptomic data of cotton *Gossypium hirsutum* L. acc. TM-1 (TM-1) plants. Since gene expression is closely related to *cis*-acting elements, we mapped the tissue-specific expression patterns of *GhVIMs* in abiotic stresses such as salt, drought, water, heat, and cold, and in roots, stems, leaves, and fibers (Fig. 5). Integrated findings from evolutionary tree studies revealed notable variations in the expression patterns of *GhVIMs* genes within specific evolutionary branches (Fig. 5A). Additionally, despite sharing similar *cis*-acting elements, genes originating from the same evolutionary branch exhibited potential functional divergences under distinct non-biological stress conditions (Fig. 5B). Collectively, the expression analysis of *GhVIMs* genes across different non-biological stressors and diverse tissue types underscores their vital significance in the intricate processes of plant growth and developme.

### Expression analysis of *GhVIMs* genes by qRT-PCR

**Fig. 6:**
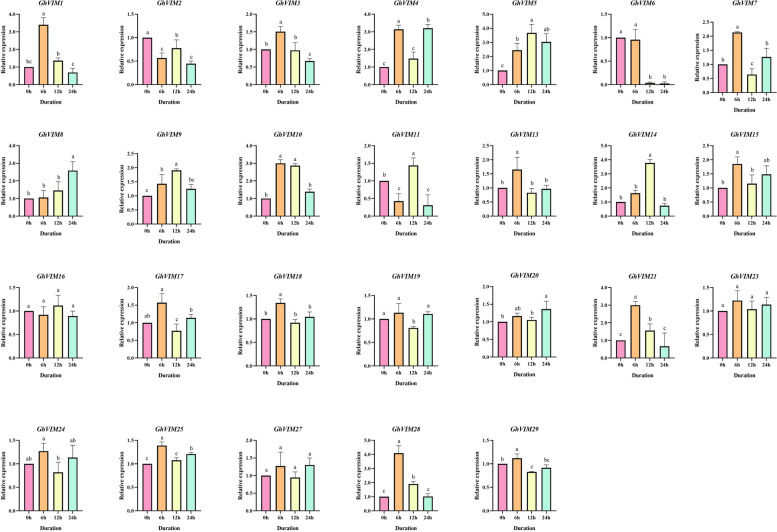
Relative expression of 26 *GhVIMs* members under salt stress at different time periods

To gain deeper insights into the expression profiles of *GhVIMs* genes in response to salt stress at different time intervals, a comprehensive analysis was conducted on 29 *GhVIMs* genes from the upland cotton evolutionary branch. However, it should be noted that three upland cotton genes, namely *GhVIM12*, *GhVIM22*, and *GhVIM26*, may have exhibited insufficient expression levels under salt stress and were inconclusive. The investigation unveiled divergent expression patterns among the 26 *GhVIMs* genes at different time points under salt stress conditions (Fig. 6). Relative expression analysis of the 26 upland cotton *GhVIM26* genes revealed that the majority of genes displayed higher relative expression levels at 6 h. For instance, *GhVIM1*, *GhVIM3*, *GhVIM4*, *GhVIM7*, *GhVIM21*, and *GhVIM28* exhibited notable increases in relative expression levels at this time point. Particularly, *GhVIM28* demonstrated the highest relative expression level at 6 h after salt stress, gradually declining at 12 h and 24 h. Conversely, the *GhVIM8* gene showed varying degrees of increased relative expression levels at 6 h, 12 h, and 24 h after salt stress. These findings highlight the distinctive characteristics and diverse responses of different genes within GhVIMs gene family when confronted with salt stress.

Error bars are the standard deviation (SD) of three biological replicates in each treatment group. Multiple comparisons were used for statistical tests. Different letters indicate significant differences between means as determined using ANOVA and LSD multiple comparisons (*P* < 0.05).

### Gene structure and protein analysis of *GhVIM28*

According to the protein physicochemical analysis, GhVIM28 has a theoretical relative molecular weight of 74.178 kDa and a theoretical isoelectric point of 7.12. The total number of negatively charged residues and positively charged residues in GhVIM28 is 98, indicating that the protein molecule is electrically neutral at its isoelectric point, with a molecular formula of C_3201_H_5085_N_941_O_1009_S_40_. The fatty acid coefficient of GhVIM28 amino acids is 61.32, suggesting its thermal stability, and the average hydrophilicity is -0.745, indicating that it is a hydrophilic protein (Figure S1A).

Signal peptide prediction indicated that GhVIM28 does not contain a signal peptide, and is a non-secreted protein (Figure S1B). Transmembrane structure analysis suggested that GhVIM28 lacks a transmembrane structure, indicating that this protein is not a transmembrane protein (Figure S1C). Secondary structure prediction of GhVIM28 showed that alpha helix and random coil are the dominant structures, accounting for 31.88% and 50.98%, respectively, followed by extended strand at 12.63%, and beta turn at 4.51% (Figure S1D). The three-dimensional structure of GhVIM28 was predicted by SWISS-MODEL homology modeling, which also showed that the main structures were alpha helix and random coil, consistent with the predicted secondary structure.

### Effect of silencing *GhVIM28* on NaCl stress in cotton

**Fig. 7:**
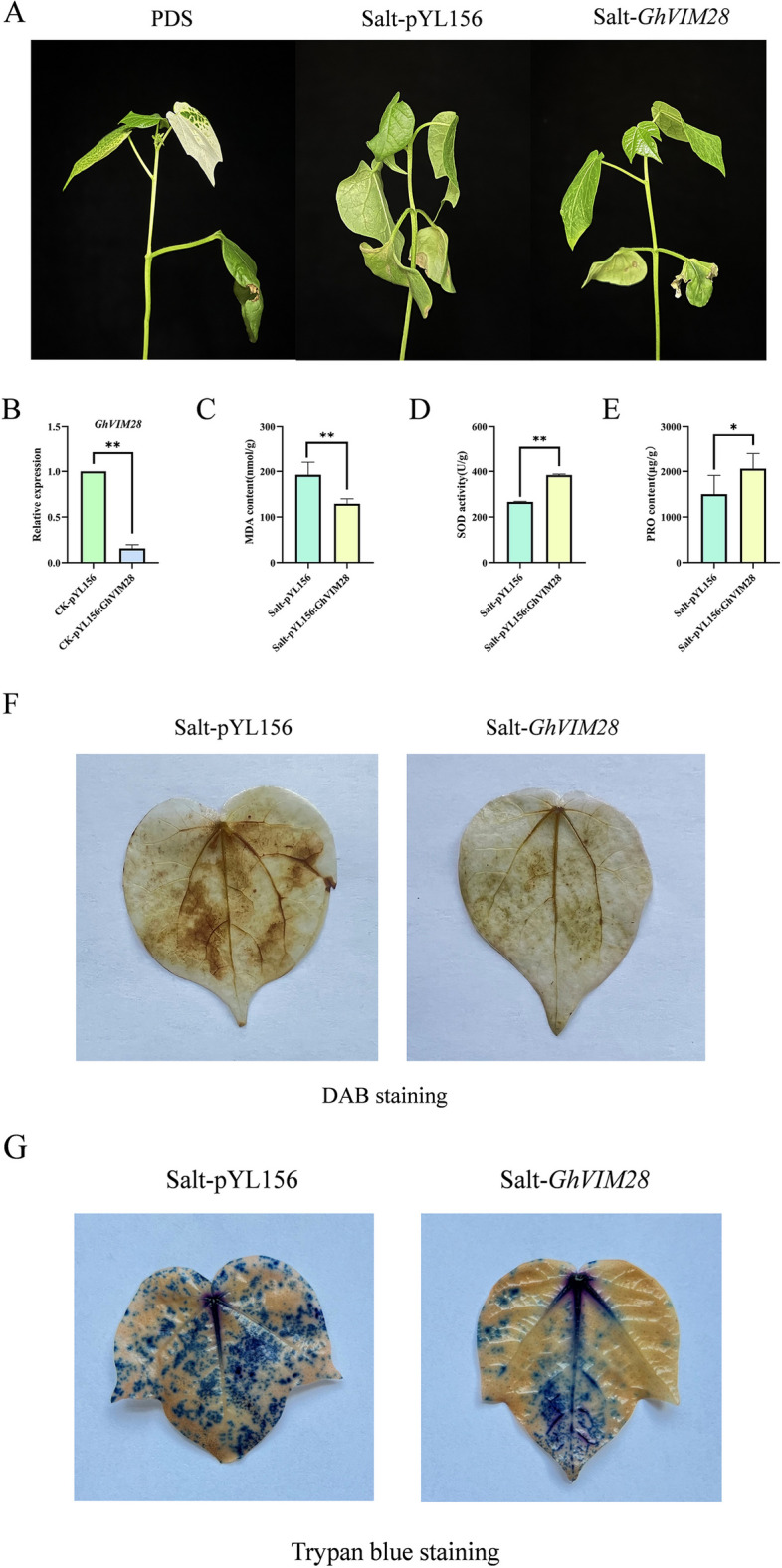
Effect of silencing *GhVIM28* on NaCl stress in cotton. **A** The phenotype of cotton after *GhVIM28* silencing under NaCl stress. pYL156: PDS as a positive control, pYL156 was an empty vector as control, and pYL156: *GhVIM28* was *GhVIM28* silenced lines. **B** Relative expression levels of *GhVIM28* in control and silenced plants. **C** MDA content of empty control and VIGS plants under NaCl stress. **D** SOD activity of empty control and VIGS plants under NaCl stress. **E** PRO content of empty control and VIGS plants under NaCl stress. **F** DAB staining. **G** Trypan blue staining *0.01 < *p* < 0.05, **p < 0.01

VIGS is a powerful tool for studying gene function [37, 38]. In order to investigate whether the *VIM* gene responded to salt stress, the *GhVIM28* gene was silenced, and the relative expression of this gene was highest at 6 h after treatment with 200 mmol/L NaCl solution, and there were significant differences in the relative expression at other time points. As shown in Fig. 7A, PDS plants showed albino symptoms, indicating successful gene silencing. Cotton leaves injected with pYL156 and exposed to 200 mmol/L NaCl treatment showed signs of dehydration and wilting compared to pYL156:*GhVIM28*. The expression level of *GhVIM28* in cotton leaves was detected by qRT-PCR, and the results, as shown in Fig. 7B, indicated that the expression level of *GhVIM28* in the CK-pYL156:*GhVIM28* group was significantly decreased compared with that in the CK-pYL156 group, indicating that *GhVIM28* was effectively silenced. In addition, after NaCl stress, the MDA content of silenced plants was significantly reduced compared with pYL156-treated plants. On the contrary, SOD activity and PRO content were significantly increased in silenced plants compared with pYL156-treated plants, as shown in Fig. 7, indicating that the antioxidant capacity of *GhVIM28* was enhanced after silencing. Therefore, it can be concluded that *GhVIM28* is a negative regulator of salt tolerance in cotton. After NaCl stress, no dark brown spots appeared on the leaves of *GhVIM28* silenced plants after DAB staining, indicating that *GhVIM28* was more lightly harmed after silencing (Fig. 7F). Cotton leaves were stained with trypan blue to record the degree of damage. The results showed that leaves of all genotypes were obviously colored after exposure to stress. Leaves of pYL156 plants had darker staining and more dead cells, while pYL156:*GhVIM28* lines had lighter coloring (Fig. 7G), This suggests that the *GhVIM28* silenced plants suffered less damaged by salt stress than pYL156 plants.

## Discussion

Through previous studies, Kraft et al. discovered that ORTH / VIM proteins function as ubiquitin E3 ligases in regulating DNA methylation [16]. The E3 ubiquitin ligase genes are present in plants, animals, and bacteria. In *Arabidopsis*, five members of the VIM family were identified, with *VIM1*, *VIM4*, and *VIM5* forming a branch within the AtORTH family. At the protein level, *VIM1* shows 95% similarity to *VIM4* and 91% amino acid identity to *VIM5*, while *VIM4* and *VIM5* exhibit 99% amino acid and nucleotide identity overall [16]. In this study, 29, 29, 14, and 17 *VIM* genes were identified in *G. hirsutum*, *G. barbadense*, *G. arboreum*, *and G. raimondii*. These results indicate that the RING-type E3 ligase gene family has a relatively small number of members, similar to the identification of the ORTHRUS / VIM family in *Arabidopsis*, which belongs to a small family. Unlike the comprehensive analysis conducted by Qanmber et al. [39], which identified 140 RING-type E3 ligases in *G. hirsutum*, our study specifically focuses on the functional characterization of the VIM branch of ubiquitin E3 ligases.

Motif refers to a relatively conserved DNA or protein sequence that is typically found in multiple genes or proteins. These conserved sequences often have specific biological functions, such as serving as binding sites for transcription factors or catalytic active sites for enzymes. Motif analysis can help researchers understand the function and mechanism of these sequences and can also be used to predict new gene family members and conduct evolutionary analysis [40]. In the evolutionary analysis of this study, we observed that members within the same branch exhibit similar motifs. There are noticeable differences in the number and types of conserved motifs among different branches, indicating a close evolutionary relationship among subfamily members and significant variations between subfamilies.

*Cis*-regulatory elements play an important role in plants under abiotic stress [41]. In this study, variations in the *cis*-regulatory elements of genes may influence gene expression and differentiation, with transcription factors responsive to these *cis*-acting elements being involved in regulating gene expression. The predicted results of *cis*-acting elements for the *GhVIMs* genes in upland cotton indicate their association with environmental stress, hormone response, development, and light response. It is inferred that the expression of *GhVIMs* genes may be regulated by environmental conditions such as light, plant hormones, and stress.

When analyzing the expression patterns of *GhVIMs* under various stress conditions, most *GhVIMs* were observed to exhibit an induced response to stress. However, certain *GhVIMs* did not show significant variation in expression under different abiotic stresses, suggesting that their primary functions may be focused on other domains or have been eliminated through evolution. Duplicated genes may play a critical role in adapting to the external environment during evolution and maintaining genetic system stability in the face of environmental challenges.

Through the study on the expression profile of heat stress, it was found that *GhVIMs* played a role under heat stress, and the expression level changed to different degrees (Fig. 5B). Reports indicate that E3 ubiquitin ligases play a role in heat stress, for example, AtSAP5 confers tolerance to heat stress [42]. AtPPRT1 has been reported to enhance heat stress tolerance in *Arabidopsis* [43]. HTD1 acts as a negative regulator of heat tolerance in *Arabidopsis* [44]. However, in our study, we were unable to study their effect under high temperature stress in cotton because high temperature stress would eliminate VIGS. The role of these E3 ubiquitin ligase genes in heat stress response can be explored using a transgenic system.

It was reported that 11 E3 ubiquitin ligase genes in rice (*Oryza sativa* L.) showed different expression patterns under different treatments [45]. In our study, 29 *GhVIM* genes exhibited different expression patterns under various treatments. For instance, *GhVIM2*, *GhVIM8*, *GhVIM19*, *GhVIM23*, and *GhVIM29* showed higher expression levels in stems, while *GhVIM13* and *GhVIM23* exhibited higher expression levels in roots. Moreover, these genes displayed distinct responses to different tissue stresses, suggesting their specific functions or involvement in important signaling pathways in roots. Additionally, a heat map analysis was performed to examine the expression patterns of the 29 genes under four abiotic stress conditions. Upland cotton demonstrated differential responses to the four stresses, particularly exhibiting lower overall gene expression levels under cold stress. It is speculated that *GhVIM* genes may play a negative regulatory role in the response to cold stress.

**Fig. 8:**
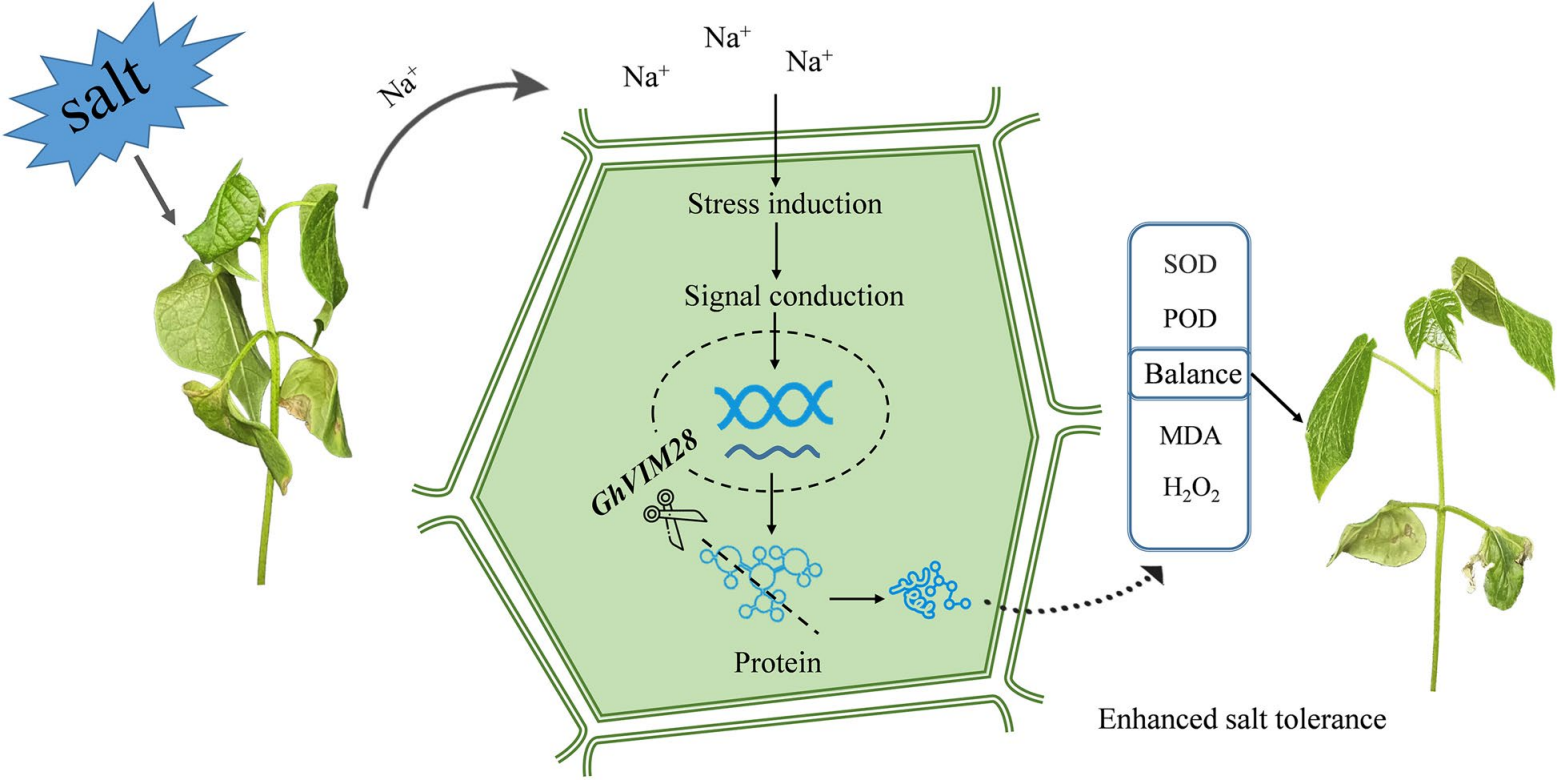
Mechanism model of *GhVIM28* responding to NaCl stress in cotton

E3 ubiquitin ligases play a crucial role in plant growth and development by regulating the biosynthesis, transport, signaling pathways, or cell cycle processes of plant hormones, participating in lateral root development in both monocotyledonous and dicotyledonous plants [46]. In *A. thaliana*, it was found that the *VIM* protein collaborates with MET1 to coordinately regulate DNA methylation and histone modification states, thereby influencing genome-wide epigenetic gene silencing [47]. The study revealed that the ubiquitination levels of two substrate complexes, ATPase subunits RPT1 (Q9SSB5) and RPT5 (Q9SEI2), were increased under salt stress, providing further evidence that the UPS system may be activated under salt stress. Additionally, significant alterations in the ubiquitination levels of certain deubiquitinating enzymes and ubiquitin-extending proteins were observed under salt stress, indicating the crucial role of the ubiquitin system in plant responses to salt stress [48]. The expression of E3 ubiquitin ligases is induced by both abiotic and biotic stresses, enhancing plant resistance. High temperature stress inhibits plant growth and seed germination, while transgenic tobacco plants overexpressing E3 ubiquitin ligases exhibit higher plant growth and seed germination than wild-type tobacco under high temperature stress, indicating their significant regulatory role under high temperature stress [46]. High temperature stress can lead to membrane destabilization in plant cells, but overexpression of E3 ubiquitin ligases can effectively protect membrane stability. In *Arabidopsis*, PUB22 and PUB23 are involved in the response to drought stress and act synergistically to negatively regulate the drought stress response. The rice SPL11 protein contains a U-box / ARM repeat structure and exhibits E3 activity. Mutations in this gene result in a phenotype with disease spots and increased resistance to certain bacterial pathogens in rice. Researchers in *Arabidopsis* have found that E3 ubiquitin ligases can be positively regulated by ABA in drought response, salt and osmotic stress responses, seed germination, and drought and salt-alkali responses. They have also been found to play a positive role in plant salt stress response, negatively regulate ethylene biosynthesis, and participate in cold tolerance [49–52]. In rice, E3 ubiquitin ligases can positively regulate plant responses to abiotic stress [53]. Silencing the *GhVIM28* gene in cotton using the VIGS system resulted in increased proline content and superoxide dismutase (SOD) activity, as well as decreased malondialdehyde (MDA) content in the plants. These findings suggest that *GhVIM28* may negatively regulate salt tolerance in cotton by modulating proline signaling transduction. Therefore, *GhVIM28* is identified as a negative regulator of salt tolerance in cotton and could serve as a novel target gene for developing salt-tolerant cotton varieties. These results further confirm previous reports that E3 ubiquitin ligases positively and negatively regulate resistance to biotic stress [45, 54–56]. In conclusion, we hypothesized the model diagram of *GhVIM28* response to salt stress (Fig. 8). *GhVIM28* gene may participate in cotton protein degradation processes, thereby triggering the antioxidant system to alleviate salt stress and promote normal plant growth. Among various forms of proteins, protein ubiquitination is widely involved in protein degradation processes, contributing significantly to several molecular mechanisms in plants, such as countering external stress through the antioxidant system. When plants with silenced *GhVIM28* gene are exposed to salt stress, they regulate the levels of proline and superoxide dismutase to eliminate the generated ROS in the body, thereby mitigating the damage of salt stress to plant cells. It has been shown in the literature that the ion stress and osmotic stress caused by salt pollution can be alleviated by osmotic regulating substances such as proline.

## Conclusion

In this study, the *VIMs* were characterized based on the results of phylogenetic relationships, gene chromosome localization and *cis*-acting element analysis. Based on the research on the cotton VIM gene family, it was found that the *VIM* genes are mainly divided into three subgroups, with the C subgroup members playing a dominant role in cotton, especially in upland cotton. Furthermore, the expression patterns of *GhVIMs* under various abiotic stresses were studied, and the function of *GhVIMs* in response to salt stress was investigated using VIGS technology. Finally, through qRT-PCR analysis and VIGS experiments on *GhVIMs* genes under salt stress conditions, it was discovered that the *GhVIM28* gene plays a negative regulatory role in cotton salt tolerance. Silencing of the *GhVIM28* gene leads to enhanced plant antioxidant capacity, thereby increasing tolerance to salt stress. This study reveals the significant role of the GhVIMs gene family in cotton growth, development, and stress responses, providing a foundation for a deeper understanding of cotton’s stress resistance.

### Supplementary Information

Supplementary Material 1.

Supplementary Material 2.

Supplementary Material 3.

## Data Availability

The genome sequence and annotation files of *G. hirsutum* L. *acc* TM-1 were obtained from COTTONMICS website (http://cotton.zju.edu.cn/index.htm). RNA-Seq data in this study have been deposited at the National Center of Biotechnology Information (http://www.ncbi.nlm.nih.gov/) under the accessions PRJNA490626 (BioProject number: PRJNA490626, SRA number: SRP166405). Zhong 9807 cotton seeds were provided by Dr. Wuwei Ye from Institute of Cotton Research of Chinese Academy of Agricultural Sciences in China.
