# Supplementary material for: GhVIM28, a negative regulator identified from VIM family genes, positively responds to salt stress in cotton
Source: BMC Plant Biol. 2024 May 21;24:432. doi: 10.1186/s12870-024-05156-8 (PMC11107009; doi:10.1186/s12870-024-05156-8)
Supplement: Supplementary file 2 — Supplementary Material 2. [file 12870_2024_5156_MOESM2_ESM.docx]

Figure S1. Bioinformatics analysis of *GhVIM28*

Figure S1
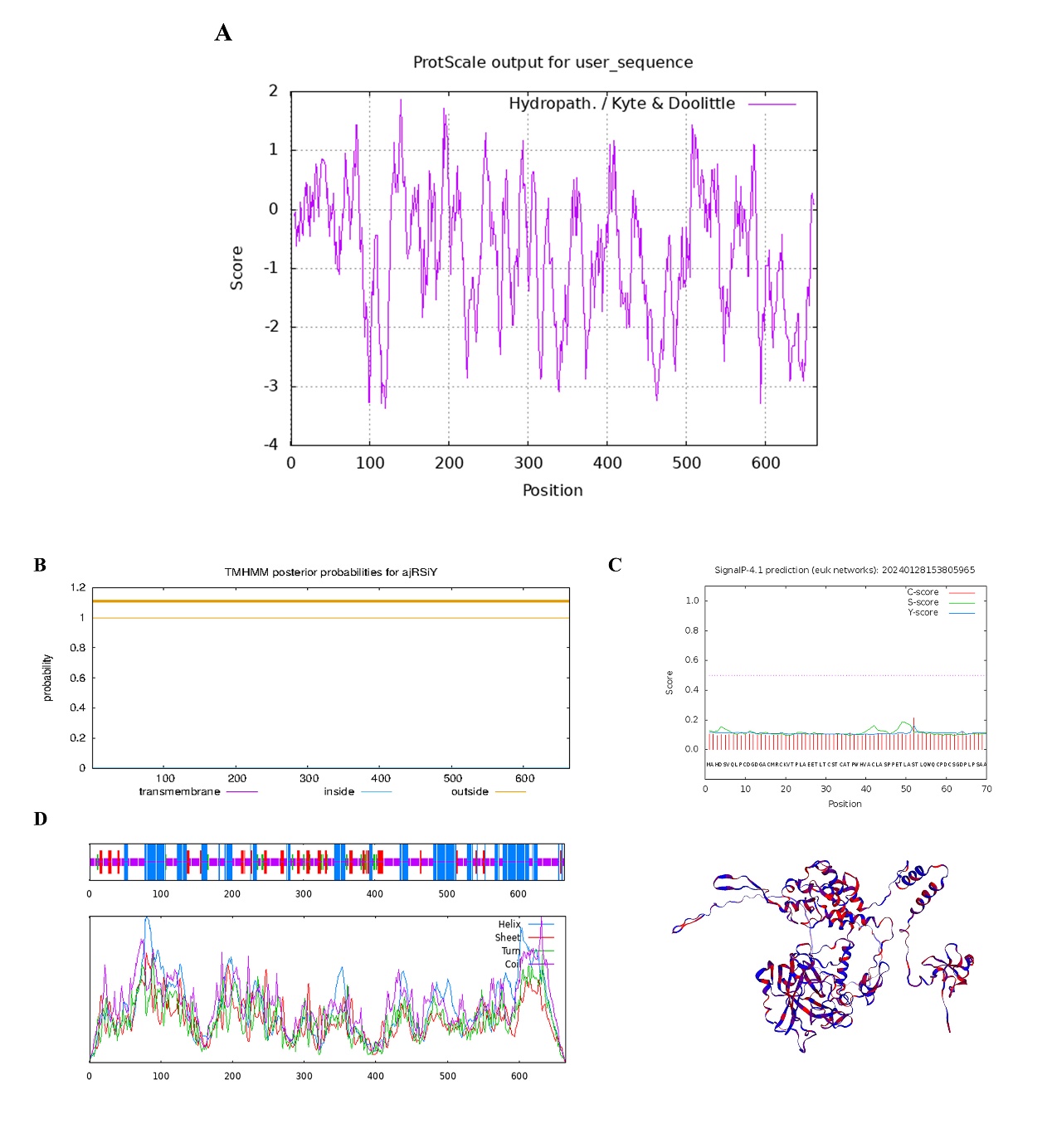
 Bioinformatics analysis of *GhVIM28*

A Hydrophilic analysis prediction. B Prediction signal peptide. C Transmembrane structure prediction. D Secondary structure and tertiary structure prediction.
