# Supplementary material for: GhVIM28, a negative regulator identified from VIM family genes, positively responds to salt stress in cotton
Source: BMC Plant Biol. 2024 May 21;24:432. doi: 10.1186/s12870-024-05156-8 (PMC11107009; doi:10.1186/s12870-024-05156-8)
Supplement: Supplementary file 3 — Supplementary Material 3. [file 12870_2024_5156_MOESM3_ESM.docx]

**Supplementary Figure S2. The map and sequence of pYL156 vector**

1. Construction of VIGS experimental vector and cloning strategy

The VIGS plasmid pYL156 was subjected to restriction endonuclides BamH I and Sac I. Subsequently, it was attached to the synthesized 300bp fragment using a one-step cloning kit (C112, Vazyme). The pYL156 plasmid map is shown in supplementary Figure 1. The constructed recombinant expression vectors were transformed into *Escherichia coli* (DH5α active cells) and *Agrobacterium tumefaciense* (LBA4404 active cells) respectively.

pYL156 vector sequence,

ATAAAACATTGCACCTATGGTGTTGCCCTGGCTGGGGTATGTCAGTGATCGCAGTAGAATGTACTAATTGACAAGTTGGAGAATACGGTAGAACGTCCTTATCCAACACAGCCTTTATCCCTCTCCCTGACGAGGTTTTTGTCAGTGTAATATTTCTTTTTGAACTATCCAGCTTAGTACCGTACGGGAAAGTGACTGGTGTGCTTATCTTTGAAATGTTACTTTGGGTTTCGGTTCTTTAGGTTAGTAAGAAAGCACTTGTCTTCTCATACAAAGGAAAACCTGAGACGTATCGCTTACGAAAGTAGCAATGAAAGAAAGGTGGTGGTTTTAATCGCTACCGCAAAAACGATGGGGTCGTTTTAATTAACTTCTCCTACGCAAGCGTCTAAACGGACGTTGGGGTTTTGCTAGTTTCTTTAGAGAAAACTAGCTAAGTCTTTAATGTTATCATTAGAGATGGCATAAATATAATACTTGTGTCTGCTGATAAGATCATTTTAATTTGGACGATTAGACTTGTTGAACTACAGGTTACTGAATCACTTGCGCTAATCAACATGGGAGATATGTACGATGAATCATTTGACAAGTCGGGCGGTCCTGCTGACTTGATGGACGATTCTTGGGTGGAATCAGTTTCGTGGAAAGATCTGTTGAAGAAGTTACACAGCATAAAATTTGCACTACAGTCTGGTAGAGATGAGATCACTGGGTTACTAGCGGCACTGAATAGACAGTGTCCTTATTCACCATATGAGCAGTTTCCAGATAAGAAGGTGTATTTCCTTTTAGACTCACGGGCTAACAGTGCTCTTGGTGTGATTCAGAACGCTTCAGCGTTCAAGAGACGAGCTGATGAGAAGAATGCAGTGGCGGGTGTTACAAATATTCCTGCGAATCCAAACACAACGGTTACGACGAACCAAGGGAGTACTACTACTACCAAGGCGAACACTGGCTCGACTTTGGAAGAAGACTTGTACACTTATTACAAATTCGATGATGCCTCTACAGCTTTCCACAAATCTCTAACTTCGTTAGAGAACATGGAGTTGAAGAGTTATTACCGAAGGAACTTTGAGAAAGTATTCGGGATTAAGTTTGGTGGAGCAGCTGCTAGTTCATCTGCACCGCCTCCAGCGAGTGGAGGTCCGATACGTCCTAATCCCTAGGGATTTAAGGACGTGAACTCTGTTGAGATCTCTGTGAAATTCAGAGGGTGGGTGATACCATATTCACTGATGCCATTAGCGACATCTAAATAGGGCTAATTGTGACTAATTTGAGGGAATTTCCTTTACCATTGACGTCAGTGTCGTTGGTAGCATTTGAGTTTCGCAATGCACGAATTACTTAGGAAGTGGCTTGACGACACTAATGTGTTATTGTTAGATAATGGTTTGGTGGTCAAGGTACGTAGTAGAGTCCCACATATTCGCACGTATGAAGTAATTGGAAAGTTGTCAGTTTTTGATAATTCACTGGGAGATGATACGCTGTTTGAGGGAAAAGTAGAGAACGTATTTGTTTTTATGTTCAGGCGGTTCTTGTGTGTCAACAAAGATGGACATTGTTACTCAAGGAAGCACGATGAGCTTTATTATTACGGACGAGTGGACTTAGATTCTGTGAGTAAGGTTACCGAATTCTCTAGAAGGCCTCCATGGGGATCCGGTACCGAGCTCACGCGTCTCGAGGCCCGGGCATGTCCCGAAGACATTAAACTACGGTTCTTTAAGTAGATCCGTGTCTGAAGTTTTAGGTTCAATTTAAACCTACGAGATTGACATTCTCGACTGATCTTGATTGATCGGTAAGTCTTTTGTAATTTAATTTTCTTTTTGATTTTATTTTAAATTGTTATCTGTTTCTGTGTATAGACTGTTTGAGATCGGCGTTTGGCCGACTCATTGTCTTACCATAGGGGAACGGACTTTGTTTGTGTTGTTATTTTATTTGTATTTTATTAAAATTCTCAACGATCTGAAA


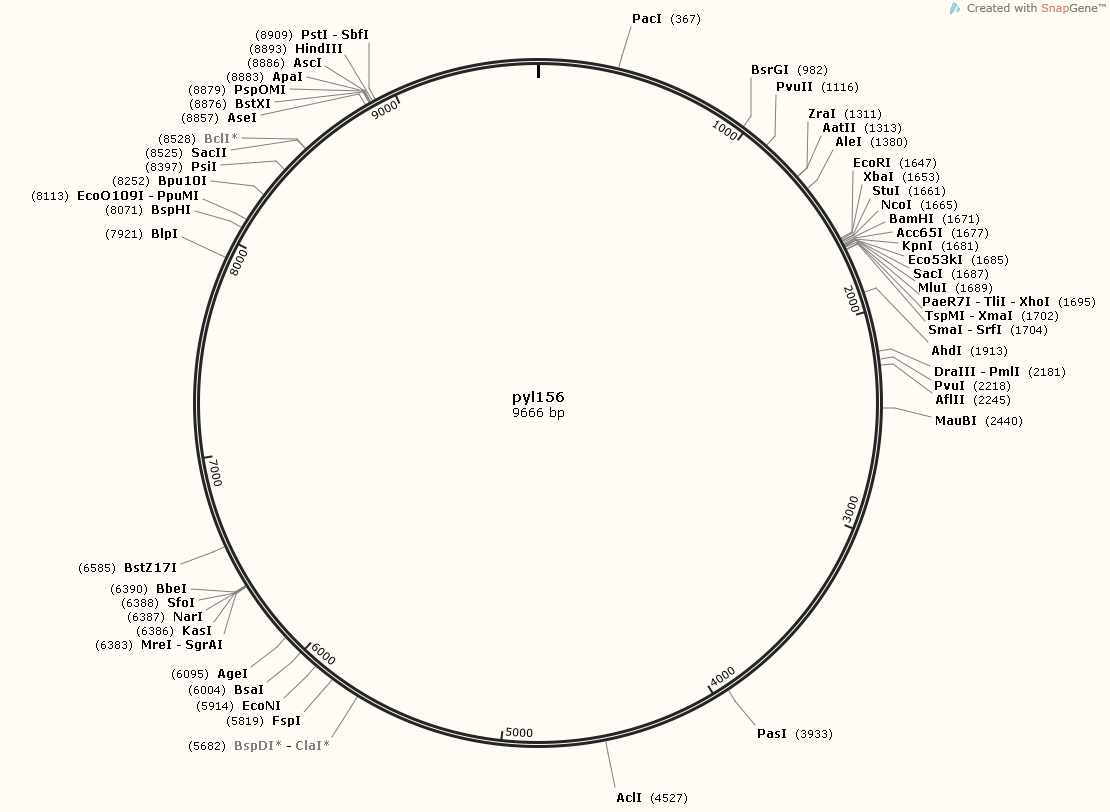


Supplementary Figure S2 pYL156 plasmid map

pYL156 is modified tobacco rattle virus RNA 2, used for virus induced gene silencing in plants along with pYL192 (TRV RNA1).
